# Research into *Neospora caninum*—What Have We Learnt in the Last Thirty Years?

**DOI:** 10.3390/pathogens9060505

**Published:** 2020-06-23

**Authors:** Michael P. Reichel, Lloyd C. Wahl, John T. Ellis

**Affiliations:** 1 Jockey Club College of Veterinary Medicine and Life Sciences, City University of Hong Kong, Kowloon, Hong Kong, China; lloyd.wahl@cityu.edu.hk; 2 Department of Population Medicine and Diagnostic Sciences, College of Veterinary Medicine, Cornell University, Ithaca, NY 14853-6401, USA; 3 School of Life Sciences, University of Technology Sydney, P.O. Box 123, Broadway, NSW 2007, Australia; John.Ellis@uts.edu.au

**Keywords:** *Neospora caninum*, dogs, cattle, abortions, diagnosis, pathogenesis, review

## Abstract

Background: *Neospora caninum* has been recognised world-wide, first as a disease of dogs, then as an important cause of abortions in cattle for the past thirty years. Over that time period, there have been improvements in the diagnosis of infection and abortion, new tests have been developed and validated, and it is timely to review progress to date. Methods: Bibliometric methods were used to identify major trends and research topics present in the published literature on *N. caninum*. The tools used were SWIFT-Review, VOSviewer and SciMAT, along with the published papers found in the MEDLINE, Dimensions and Web of Science databases. A systematic review of the published *Neospora* literature (*n* = 2933) was also carried out via MEDLINE and systematically appraised for publications relevant to the pathogenesis, pathology and diagnosis of *Neospora* abortions. Results: A total of 92 publications were included in the final analysis and grouped into four main time periods. In these four different time periods, the main research themes were “dogs”, “abortion”, “seroprevalence” and “infection”. Diagnostics, including PCR, dominated the first two time periods, with an increased focus on transmission and abortions, and its risk factors in cattle. Conclusions: Longitudinal analyses indicated that the main themes were consistently investigated over the last 30 years through a wide range of studies, with evolving emphasis initially on dogs and diagnostic test development, followed by application to cattle, the identification of the risk factors leading to abortion, and in the latter time periods, an understanding of the immunity and a search for vaccines.

## 1. Introduction

*Neospora caninum* is a protozoan parasite that was first recognised about 30 years ago as the aetiological cause of abortion in infected cattle [1,2,3]. *N. caninum* has since become one of the most frequently diagnosed causes of abortions in cattle and is estimated to cause cattle producers world-wide losses exceeding a billion dollars annually [4]. Seroprevalence studies show infection rates vary considerably between and within countries, and between dairy and beef cattle [4]. For example, recovery rates of *N. caninum* from aborted cattle foetuses range from as low as 12% to as high as 42% [5]. There is evidence that *N. caninum* may cause reproductive disruption in small ruminant species, such as sheep and goats [6,7]. There have also been reports of cerebral neosporosis in sheep [8]. The water buffalo (*Bubalus bubalis*) appears to be readily infected with *N. caninum*, however abortions in this bovid are rarely reported [9]. Dogs are the primary definitive host for this parasite in which sexual replication occurs [10], and they may also be affected clinically [11,12]. Other canids may also act as definitive hosts in parts of the world [13,14].

While we have known *N. caninum* to be a cause of infection and abortions in cattle for thirty years, there has been ongoing discussion about how to best diagnose and prevent a *Neospora* abortion [15], as well as the ways to develop appropriate, practical, economically justifiable and efficacious tools to prevent them. The bulk of publications on the subject is approaching 3000 scientific manuscripts and these present our combined knowledge on the subject. The following presents an analysis of that literature of those three decades in a systematic way, identifies the key steps in our understanding, and documents the progress of the past three decades as well as possible steps for the future.

## 2. Results

Four different databases were searched for literature using the term “*Neospora*” (Table 1). All databases returned slightly different numbers of papers depending on the search performed. Although a search of Web of Science (WoS) with the term “*Neospora*” returned the highest number of publications, MEDLINE provided a higher number of papers relating to terms of importance to this review (diagnosis, pathology and pathogenesis).

### 2.1. Topic Modelling

**Figure 1:**
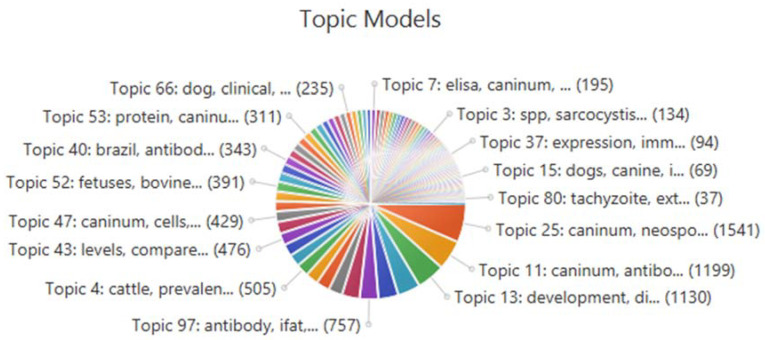
Overview of topic models generated from the MEDLINE dataset using SWIFT-Review.

Topic modelling of the literature from MEDLINE on “*Neospora*” was performed in SWIFT-Review [16]. This approach is helpful in the identification of the major themes present in the literature through the identification of key words included in the topic model. The top 15 topic models (of the 100 returned) are presented in Table 2 and Figure 1. Table 2 includes lay descriptions formulated as an attempt to provide a simple meaning to the topic model as a theme. Most of the topics identified are associated with diagnosis and pathology of infections in cattle and experimental infections in mice. Serology, seroprevalence, molecular diagnostics, disease development and risk analyses are the key themes that dominate.

### 2.2. Bibliometric Analysis

**Figure 2:**
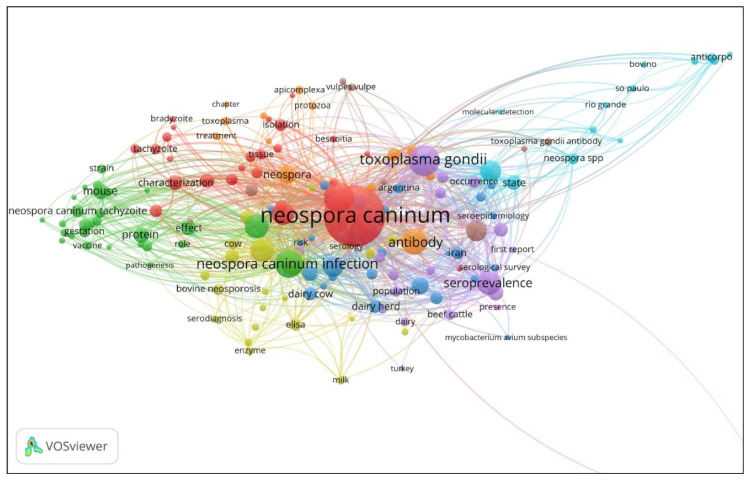
VOSviewer network with nine colour-coded clusters produced using VOSviewer and the Dimensions dataset containing 2935 unique publications from 1989 to 2019 inclusive (for clarity, links to the terms “Colombia” and “seroprevalencia” have been truncated at the bottom right hand corner).

A search of the Dimensions database using the term “*Neospora*” returned 2987 publications between 1989 and 2019; references with identical titles were deleted, leaving 2935 publications, which were imported into VOSviewer (www.vosviewer.com, Universiteit Leiden, Leiden, Netherlands, Version 1.6.15) [17] for bibliographic analyses. The main network characteristics obtained from an analysis of co-occurrence of words are shown in Table 3 and Figure 2. Of the 5571 terms present in the paper titles, 167 major terms (nodes) met the minimum occurrence of 10 specified. This data is organised by VOSviewer into nine main clusters, which are summarised in Table 3. These nine clusters represent simply a high-level overview of the main studies that were carried out on *Neospora*. Some are easy to identify in terms of lay description; for example, cluster 2 is primarily about using mouse models to study vaccine development and drug treatment. At least four of the clusters are closely linked to the use of serology in the study of reproductive performance, epidemiology and seroprevalence (clusters 3–5 and 9). Cluster 6 relates to the application of molecular diagnostics to define the disease burden in Brazil; and cluster 8 points to studies on the red fox (*Vulpes vulpes*) as a host.

### 2.3. SciMAT

**Figure 3:**
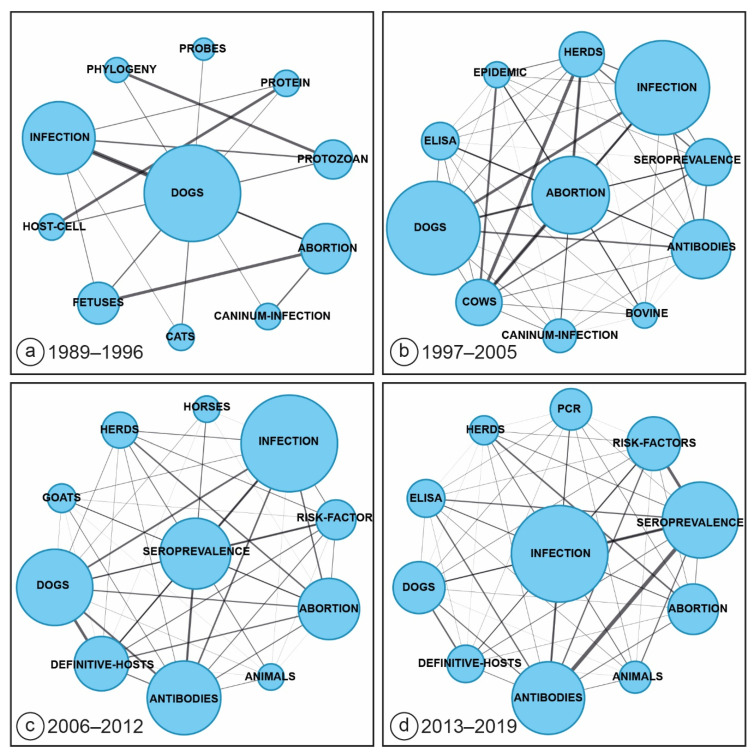
Main themes in *Neospora*-related publications from the Web of Science (WoS) over four time periods identified using SciMAT ((**a**)—1989–1996 (*n* = 15); (**b**)—1997–2005 (*n* = 733); (**c**)—2006–2012 (*n* = 1172) and (**d**)—2013–2019 (*n* = 1013)). The figure shows the links in terms to the four major themes identified during these time periods.

The identification of *N. caninum* in dogs [10] and, subsequently, as a cause of abortion in cattle [2] was a significant milestone in *N. caninum* research. This is reflected in the longitudinal study performed using SciMAT and the WoS dataset (3470 publications); between 1989 and 1996 (in a dataset of *n* = 15 publications) the term “dogs” is commonly used in association with terms such as “abortion, fetuses and infection”. PCR emerges as a common technology being used in these studies. In the period of 1997–2005 (*n* = 733 publications), “abortion” emerges as the main motor theme and seroprevalence studies of herds using Enzyme-linked immunosorbent assays (ELISA) technology is recognized through association. During 2006–2012 (1172 publications), “seroprevalence” becomes the main motor theme identified by SciMAT, which is associated with words such as “risk factors, animals, goats and horses”. In the 2013–2019 (*n* = 1013) period, “infection” (which has been a feature as a secondary theme throughout the time periods) is now the major motor theme in association with most of the topics identified previously including dogs, animals, herds, risk factors, abortion, antibodies, PCR and seroprevalence (Figure 3.).

### 2.4. Systematic Review of Literature

**Figure 4:**
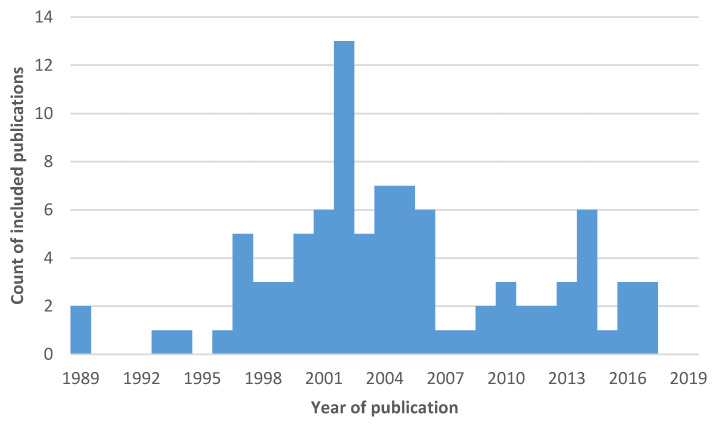
Frequency histogram of 92 publications derived from a systematic search of the *Neospora* literature, conducted in March 2020 in the Dimensions database for search terms: *Neospora*, dogs, abortion, seroprevalence and infection; showing peak publication activity between 1996 and 2006, with the highest number of publications in the search yielding from the year 2002.

The systematic literature analysis, guided by SciMAT, yielded a reduced literature field of a total of 92 scientific manuscripts meeting the citation criteria: five publications with citation ratios above 5 were included for the 1989–1996 (“dog”) time period; for the 1997–2005 (“abortion”) period, 54 publications; for the 2006–2012 period (“seroprevalence”) 17 publications, and 16 publications for the 2013–2019 (“infection”) period (Figure 4.).

#### 2.4.1. Time Period 1989 to 1996 (*N. caninum* and Dog)

The five publications extracted from the SciMAT-guided literature review yielded three publications that described the establishment, validation and use of serological techniques for the detection of *N. caninum* infection in dogs, such as Immunohistochemistry (IHC) [1], Enzyme-linked immunosorbent assays (ELISA) [18] and Indirect Fluorescent Antibody Test (IFAT) [18,19]. One study used the IFAT as a diagnostic criterion for establishing *N. caninum* infection in dogs and described the clinical aspects of a number of cases [12], while the other used IFAT to describe the prevalence of infection in a population of dogs in the United Kingdom [19].

One of the included studies also confirmed, via experimental infection that *N. caninum* can be passed from bitch to pup in utero [11].

#### 2.4.2. Time Period 1997 to 2005 (*N. caninum* and Abortion)

This time period yielded the highest total (54) of publications from the literature review and more than 50% of the overall total included in the final literature appraisal, as well as the highest number of included publications (*n* = 13) from a single year (2002). The majority of publications in that time period focused on an understanding of the transmission of the parasite, control and its epidemiology, while a considerable number of manuscripts elaborated on diagnostic test development, validation and their application.

Varying technologies were used in establishing and validating serological tests; immunostimulating complexes (ISCOM) [20] and avidity ELISA [21], and the use of purified [22], even recombinant antigens [23], the application to different testing media, such as milk [24] and different test modalities [25], as well as adjustments to cut-off thresholds in the assays [26]. Immunoblot analysis described reaction patterns that could be used in the diagnosis [27]. Other diagnostic tools, such as histopathology, IHC and PCR were evaluated and validated [28,29,30,31]. The severity of fetal lesions contributing to abortions was also assessed [32].

Our knowledge on the transmission of the parasite in cattle populations was enhanced by an understanding of the relative importance of vertical [33,34,35,36,37] versus post-natal transmission [38,39,40,41,42,43], often occurring simultaneously in the same cattle herd [44]. Based on that a better understanding of the risk factors contributing to *N. caninum* abortions was achieved [45,46,47], and mitigation strategies suggested, such as, in one study, the use of beef semen [48].

Two studies focused on the economic losses due to *N. caninum* infection in dairy cattle production systems [49,50], concluding that milk production was significantly reduced in herds experiencing an abortion outbreak, and putting a cost estimate on production impacts per infected 50 cow herd.

Three publications explored treatment options for *N. caninum* infection, using antiprotozoal drugs, first in vitro [51], others in a mouse model [52], ultimately in cattle [53]. The latter study showed complete resolution of an experimental infection with tachyzoites of *N. caninum*.

Characterisation of a number of isolates of *N. caninum* was undertaken, in Australia [54], Portugal [55] and isolates compared to other apicomplexans [56]. *N. caninum* isolates showed themselves to be unique to other apicomplexan parasites, with genetic markers being able to be used to differentiate between different isolates.

Studies into the immunity of *N. caninum* infection were published in that time period [57,58,59]. With a better understanding of the immunological consequences of infection, subsequent efforts to produce efficacious vaccines, first in a mouse model [60], then also in the target species, cattle [61] were described. This inactivated vaccine reduced abortions in cattle by about 50%.

Further seroprevalence studies added information on *N. caninum* occurrence in cattle around the world, from Iran [62] and Brazil [63], and concluded that it rarely occurred in sheep or pigs [64].

#### 2.4.3. Time Period 2006 to 2012 (*N. caninum* and Seroprevalence)

The total of 17 publications included in this time period describe the seroprevalence of *N. caninum* infection in a number of species and countries; one study reported on the dingo (*Canis lupus dingo*), after the realisation a couple of years earlier that the dingo can also be a definitive host for *N. caninum* in Australia [14]. This study [65] found a high (27.0%) seroprevalence in wild and aboriginal dogs in indigenous communities in Australia, as well as some oocysts.

Another intermediate host was determined in a study on chickens’ ability to host *N. caninum* [66], and a high rate of serological reactions was determined in ravens (*Corvus corax*) [67].

Several studies described the seroprevalence and risk factors of *N. caninum* infection in cattle populations (beef and dairy), across a number of countries and continents; such as Spain [68,69], Senegal (West-Africa) [70], North [71,72] and South America [73], as well as a multi-national study from Europe showing wide differences in individual and herd-level infection rates [74]. Risk factors repeatedly identified included the presence of dogs and the feeding of colostrum. Seroprevalence of *N. caninum*, in general, was lower in beef than in dairy cattle.

An additional study, from Brazil, established infection rates in sheep [75].

Several studies focused on *N. caninum* infection in wildlife, such as wild foxes and deer in Europe [76,77], various wildlife species in Alaska [78] or the USA [79], and zoo populations in Europe [80].

One study simulated four different control strategies for *N. caninum*, including maintaining the *status quo* (doing nothing), test and cull, treatment and vaccination [81], concluding that complete eradication was not possible if horizontal transmission of the parasite could not be curtailed.

#### 2.4.4. Time Period 2013 to 2019 (*N. caninum* and Infection)

The 16 publications group into several topic areas; studies of the prevalence and risk factors for *N. caninum* infection for different species in different parts of the world, as varied as dogs [82], cats [83], cattle [84], small ruminants [85,86,87], horses [88] and wildlife [89].

More general investigations on the immune responses after an *N. caninum* infection were described in sheep as a model of infection [90], and in cattle of specific antibodies as a predictor of abortion [91], as well as the importance of the specific isolate that causes infection leading to abortion in cattle [92], and in cattle [93] and dogs [94] on Neutrophil Extracellular Traps (NET) Release.

A further publication explored calcium-dependent protein kinases as drug targets for apicomplexans, including *N. caninum* [95], another utilised an in silico approach to identify potential drug candidates against *N. caninum* [96].

#### 2.4.5. All Time Periods

**Figure 5:**
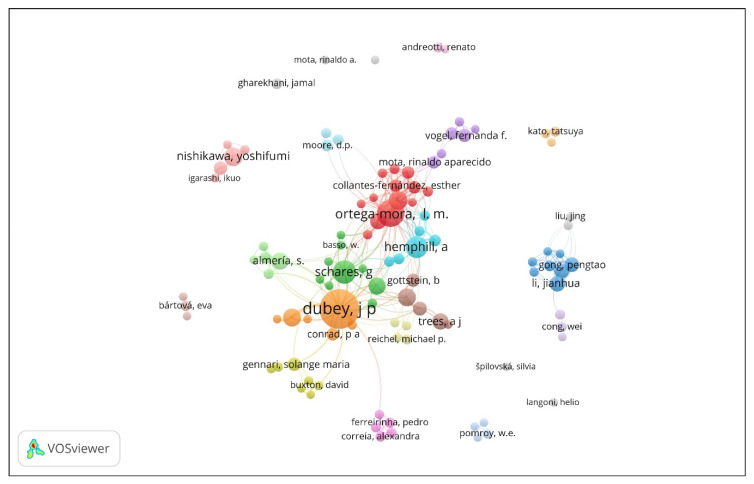
Author network from 1107 *N. caninum* publications from targeted SciMAT process.

While this systematic literature search covered seminal publications that indicate, and likely influenced the direction that *N. caninum* research took over the past three decades, each of those directions is indicative of a larger body of scientific work that developed along similar lines. An author network from 1107 publications from Dimensions demonstrates clearly a central, well interlinked node of ~125 authors in the *N. caninum* scientific literature, along a (North and South) American continent–Europe centric axis, with peripherally several smaller nodes in places such as New Zealand, Australia, China and Japan contributing to the significant literature on *N. caninum* from around the world (Figure 5).

## 3. Discussion

The scientific literature on *N. caninum* over the past 30 years developed after the initial recognition of the infection in dogs in the late 1980s. Topical modelling of the relevant literature describes *N. caninum* research over the past three decades focussed on abortions in cattle, the validation of various diagnostic tests and the application of these tests to the investigation of the epidemiology of infection, and the risk factors contributing to abortion events. VOSviewer analysis and word clusters develop similar themes around the diagnosis and epidemiology of abortions due to *N. caninum* in cattle. These advances allowed us to diagnose abortions in cattle accurately, establish that two quite different modes of transmission of the infection are observed and what risk factors might contribute to abortion (epidemic) events and should, therefore be mitigated.

The SciMAT-guided systematic literature review identified a separate guiding theme for four distinct time periods; the first time period from 1989 to 1996 focused on *N. caninum* and dogs. Diagnostic tests for *N. caninum* infection in dogs were first described and validated, and the experimental studies confirmed that infection can be vertically transmitted in dogs.

In the early parts of the subsequent time period (1997–2005), came the confirmation that dogs are one definitive host of *N. caninum* in which the sexual reproduction of the parasite is completed [10] Other canids, such as coyotes [13] were also identified as potential definitive hosts. Further on in that period. which was also the most productive in terms of the number of highly regarded publications, the focus soon switched to cattle, with the realisation of *N. caninum* as a significant cause of abortion in that species. That time period then concentrated on diagnostic test development and validation, and their application to populations of cattle around the globe. The identification of two types of transmission modalities in cattle, vertical and post-natal, led to an increased interest in epidemiological studies, describing these two modalities, as well as risk factors for either of these two modes to be realised. The immunological events and the timing of infection driving either outcomes in cattle (abortion or congenital infection) were studied [97]. A better understanding of the underlying immunology led to a search for and application of vaccines, although early vaccines had limited efficacy in preventing abortion [61] and have subsequently been withdrawn from the market.

In the third examined time period (2006 to 2012), there was an increased interest in other possibly *N. caninum* affected species, such as wildlife, birds, small ruminant populations, as well as further research on prevalence studies around the world, enabling a better understanding of the prevalence and epidemiology (possible wildlife reservoirs, incl. the identification of the dingo in Australia as a definitive host species [14]) as well as a simulation of efficacious and economic control strategies. A better understanding of the parasite-cattle interaction led to experimental vaccines that were 100% efficacious [98].

The most recent time period (to 2019), continues to explore additional host species (wildlife, small ruminants) and explores a more sophisticated understanding of the immunology of infection and possible drug targets in cattle.

The changing themes over the time period show progress in line with the developing needs of our understanding of the parasite and its epidemiology and how research responded to these challenges. Research improved our understanding of *N. caninum*, first with the discovery of the novel parasite in dogs, then as an abortifacient in cattle, and was aided by the development of (better) diagnostic techniques and tests. Ultimately, their broader application lead to a better understanding of its epidemiology and immunology. A better understanding of the epidemiology (incidence and prevalence) also allowed a better assessment of the economic cost of the losses incurred by cattle owners and gave rise to research into vaccines to counter these losses.

The research described here has been key to our improved understanding of the diagnosis, disease ecology and epidemiology of *N. caninum* and the immune responses to its infection. Risk factor analysis has promoted improved biosecurity measures on the farm [99]; and analysis of the economic impact of *N. caninum* [4] should have provided an incentive for the funding of future research.

This next phase of research should see a substantially increased focus on drug and vaccine development [100]. Our understanding of the parasite allows us to focus efforts on efficaciously breaking the transmission cycle with appropriate vaccines [98]; future vaccines that need to be commercially acceptable.

In silico approaches to vaccine development for *N. caninum* were proposed in recent years, including those built on the reverse vaccinology pipeline *Vacceed* [101]. Other studies highlighted the need for improved genome assemblies [102] and detailed annotation of the *N. caninum* genome [103]. It is anticipated that genome assemblies from long and short read sequencing technology will lead to new advances in the application of reverse vaccinology to *N. caninum* [104] and ultimately efficacious vaccines for that parasite.

Drug development needs to focus on economically viable treatments, that are also safe. A starting point might be the re-evaluation and re-purposing of established anti-parasitic drugs, first *in-vitro* and then subsequently their evaluation in the appropriate animal models [96,105,106].

There also seems, at least occasionally, and particularly from Brazil, evidence that infection with *N. caninum* can be detected in humans, most recently *N. caninum* genetic material in the umbilical cord bloods of a small number of fetuses. About a quarter of the cord samples tested also showed IgG antibodies to *N. caninum* in the IFAT [107]. This study also suggested that an association existed between positive sero-status and exposure to dogs. This follows on from an earlier study from that country that demonstrated a high level of sero-positivity (38%) in samples from HIV infected individuals, 18% in people with neurological symptoms but also with lower percentages (5%) from newborns [108]. Other studies had previously concluded that evidence of *N. caninum* infection was not detectable in human blood sample panels [109] or responsible for reproductive failure in humans [110]. These recent observations might warrant further investigation.

## 4. Materials and Methods

Selected publications were analysed in SWIFT-Review [16]; the data from a MEDLINE (through PubMed; https://pubmed.ncbi.nlm.nih.gov/) search with the term “*Neospora*” was exported to a file as a PMID list, which was then imported into SWIFT-Review for topic modelling. Since MEDLINE does not contain citation data, other databases were used as the source of bibliographic data.

The entire bibliometric data from a “*Neospora*” search (title and abstract, date accessed 25 March 2020) for the time period studied was exported from the Dimensions database as a csv file and any duplicates present identified and removed using SQL in the DB Browser for SQLite; the revised dataset was imported into VOSviewer [17]. A bibliometric analysis was performed based on co-occurrence of words in paper titles using binary counting; a minimum of 10 occurrences in the dataset was specified.

In order to document the evolution of research on *N. caninum*, the bibliometric data present in WoS (which including authors key words) for the topic “*Neospora*” (accessed on 25 March 2020) was obtained. This data was imported into SciMAT [111] for analyses; singular and plural versions of the same words were grouped. An analysis for co-occurrence of words over time was performed under varying parameters. The publications were arbitrarily assigned to four time periods (1989–1996, 1997–2005, 2006–2012, 2013–2019) so that changes over this chronological period could be investigated, using the workflow described elsewhere [112]. In summary, normalisation was performed using the equivalence index; the simple centers algorithm was used and the minimum network size was 3. Evolution and overlapping maps used the Jaccard and inclusion indices, respectively. Important motor-themes were identified by their location in the upper-right hand quadrant of the strategic diagram generated by SciMAT.

**Figure 6:**
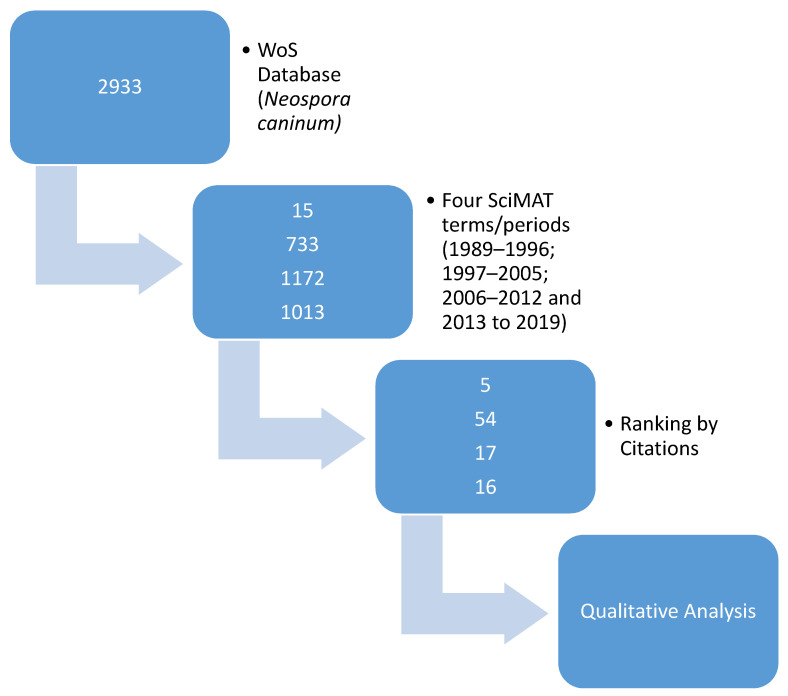
Graphical depiction of the systematic literature review process of the WoS database (numbers in the blue boxes represent the number of papers analysed at that step).

A systematic review of the total available English language peer-reviewed literature on *Neospora caninum* in the Dimensions database (accessed in 25 March 2020; 2933 publications) was carried out on the four main motor themes (e.g., Neospora AND abortion for 1997–2005) identified in SciMAT. Publications were restricted to the relevant time period related to the motor theme. Publications were assessed according to Relative Citation Ratio (RCR) for the 1989–1996 time period and Field Citation Ratio (FCR) for the other three, previously defined time periods of 1997–2005, 2006–2012 and 2013–2019. A value for the RCR or FCR of greater than 5 (as a proxy indicating peer-acknowledged quality) was used to justify inclusion of a publication for review and inclusion in this study. Reviews, editorials and Short communications were excluded from further analysis (Figure 6).

Co-authorship networks were constructed in VOSviewer using a dataset obtained from the Dimensions database. This was guided by the SciMAT outcomes and constructed for the four time periods using searches of the title and abstract for (Neospora AND dog, 1989–1996) plus (Neospora AND abortion, 1997–2005) plus (Neospora AND seroprevalence, 2006–2012) plus (Neospora AND infection, 2013–2019). This generated 1107 publications. Where multiple forms of authors names existed (e.g., Dubey, J.P., Dubey JP), they were standardised to one of the options using search/replace in Excel. Networks were constructed using fractional counting and a minimum number of publications per author of 5.

## Figures and Tables

**Table 1:** Summary of the number of papers identified in searches of different databases.

Search Terms	MEDLINE	Dimensions ^1^	Scopus ^2^	WoS ^3^	WoS ^4^
*Neospora*	2379	2905	2998	3068	3470
(“*Neospora*”) AND Diagnosis	1016	329	442	872	882
(“*Neospora*”) AND pathology	430	42	211	80	147
(“*Neospora*”) AND pathogenesis	2010	76	115	143	145

^1^ (title and abstract); ^2^ (article, title, keywords); ^3^ (topic); ^4^ (all fields). Accessed on 25 March 2020.

**Table 2:** Top 15 topic models identified by SWIFT-Review of “*Neospora*” literature from MEDLINE.

Topic Number	Topic Words	Number of Publications Contributing to Topic Model	Lay Description of Topic
25	*caninum*, *Neospora*, cattle, neosporosis, parasite, infection, abortion, bovine, important, disease	1541	*Neospora* as a cause of abortion in cattle
11	*caninum*, antibodies, *Neospora*, seroprevalence, infection, samples, study, positive, prevalence, presence	1199	Seroprevalence of *Neospora*
13	development, disease, studies, species, including, animal, diseases, vaccine, approach, understanding	1130	Disease development and control
49	*gondii*, *Toxoplasma*, caninum, *Neospora*, parasites, infections, toxoplasmosis, animals, study, species	911	*Neospora* and *Toxoplasma* infections in animals
61	*caninum*, pcr, dna, samples, *Neospora*, positive, detection, detected, brain, tissues	802	PCR detection of *Neospora* in animal tissues
97	antibody, ifat, *caninum*, test, antibodies, indirect, *Neospora*, serum, fluorescent, samples	757	Immunofluorescent antibody testing
29	*caninum*, mice, infection, *Neospora*, tachyzoites, infected, days, inoculated, parasite, experimental	709	Experimental infections in mice
27	*caninum*, *Neospora*, lesions, infection, neosporosis, brain, tachyzoites, immunohistochemical, immunohistochemistry, encephalitis	559	Immunohistochemistry and pathology
17	cows, *caninum*, seropositive, dairy, *Neospora*, abortion, seronegative, heifers, animals, cattle	540	Serology in cattle
36	risk, factors, farms, seroprevalence, study, seropositivity, analysis, regression, herds, logistic	512	Risk analyses
4	cattle, prevalence, *caninum*, seroprevalence, herds, dairy, *Neospora*, infection, beef, herd	505	Seroprevalence of *Neospora* in cattle
19	test, tests, results, sensitivity, bovine, elisa, serological, sera, specificity, cattle	489	Serological diagnostics for cattle
78	*caninum*, antigens, antigen, kDa, tachyzoites, *Neospora*, recognized, tachyzoite, antibodies, monoclonal	483	Identification of antigens
43	levels, compared, study, significant, significantly, observed, results, higher, increased, found	476	Diagnostics for cattle
33	host, parasite, cell, *Neospora*, parasites, intracellular, *caninum*, infection, apicomplexan, *Toxoplasma*	471	*Neospora* and *Toxoplasma* infections in animals

**Table 3:** Summary of the word clusters identified using VOSviewer and the Dimensions dataset obtained using a search for the term “*Neospora*”.

Cluster	Cluster/Lay Description	Keywords	Species and Diseases	Countries Mentioned
1	*Neospora caninum*	Brain, calf, isolation, bradyzoite, detection, isolate, isolation, PCR, oocyst, molecular characterisation	*Besnoitia*, *Hammondia*, *Hammondia heydorni*, *Sarcocystis*	Japan
2	Mouse models	Experimental infection, gestation, immunisation, placenta, pregnancy, protection, strain, toltrazuril, vaccine, vertical transmission	Balb/C mouse	
3	Reproduction	Abortion, association, cause, evidence, herd, reproductive performance, review, risk, seroepidemiology, serological survey	*Bubalus bubalis* (water buffalo), dairy cow, *Mycobacterium avium* subspecies *paratubuculosis*,	Argentina, Australia, Iran, New Zealand
4	Seroepidemiology	Anti *Neospora caninum* antibody, blood, cat, control, diagnosis, elisa, epidemiology, milk, serodiagnosis, transplacental transmission	bovine neosporosis, *Toxoplasma gondii* infection, toxoplasmosis	
5	Seroprevalence	dairy farm, disease, investigation, *Neospora caninum* antibody, occurrence, population, risk factor, seroprevalence, serosurvey	Beef cattle, goat, sheep, *Toxoplasma gondii*	China, Mexico, Brazil, Spain, Turkey
6	Molecular detection in Brazil	Bovino, molecular detection, state	*Neospora* spp.	Brazilian locations
7	Treatment of cyst-forming coccidia	Antibody, apicomplexan, chapter, factor, protozoa, treatment	Equine protozoal myeloencephalitis, horse, *Neospora*, *Toxoplasma*, *Sarcocystis*	Italy
8	Foxes	Parasite, prevalence, *Toxoplasma gondii* antibody	*Vulpes vulpes* (red fox),	Czech republic, Germany
9	Prevalence in Columbia	Presence, seroprevalence		Columbia
